# Combining external physical stimuli and nanostructured materials for upregulating pro-regenerative cellular pathways in peripheral nerve repair

**DOI:** 10.3389/fcell.2024.1491260

**Published:** 2024-11-06

**Authors:** Eugenio Redolfi Riva, Melis Özkan, Francesco Stellacci, Silvestro Micera

**Affiliations:** ^1^ Department of Excellence in Robotics and AI, The BioRobotics Institute, Scuola Superiore Sant’Anna, Pisa, Italy; ^2^ Bertarelli Foundation Chair in Translational Neural Engineering, Center for Neuroprosthetics and Institute of Bioengineering, École Polytechnique Federale de Lausanne, Lausanne, Switzerland; ^3^ Institute of Materials, École Polytechnique Fédérale de Lausanne (EPFL), Lausanne, Switzerland; ^4^ Department of Bioengineering and Global Health Institute, Institute of Materials, École Polytechnique Fédérale de Lausanne (EPFL), Lausanne, Switzerland

**Keywords:** nanomaterials, nerve regeneration, nir light, piezoelectric, magnetic, electrical stimulation, conductive polymers

## Abstract

Peripheral nerve repair remains a major clinical challenge, particularly in the pursuit of therapeutic approaches that ensure adequate recovery of patient’s activity of daily living. Autografts are the gold standard in clinical practice for restoring lost sensorimotor functions nowadays. However, autografts have notable drawbacks, including dimensional mismatches and the need to sacrifice one function to restore another. Engineered nerve guidance conduits have therefore emerged as promising alternatives. While these conduits show surgical potential, their clinical use is currently limited to the repair of minor injuries, as their ability to reinnervate limiting gap lesions is still unsatisfactory. Therefore, improving patient functional recovery requires a deeper understanding of the cellular mechanisms involved in peripheral nerve regeneration and the development of therapeutic strategies that can precisely modulate these processes. Interest has grown in the use of external energy sources, such as light, ultrasound, electrical, and magnetic fields, to activate cellular pathways related to proliferation, differentiation, and migration. Recent research has explored combining these energy sources with tailored nanostructured materials as nanotransducers to enhance selectivity towards the target cells. This review aims to present the recent findings on this innovative strategy, discussing its potential to support nerve regeneration and its viability as an alternative to autologous transplantation.

## 1 Introduction

Neurotmesis is the most severe among all the peripheral nerve injuries as it is characterized by extended regeneration periods and incomplete functional recovery, often resulting in long-term sequelae such as chronic pain and deficits in motor or sensory function. The associated socioeconomic and psychosocial impacts are substantial, involving prolonged absences from work, potential occupational reassignment, and significant psychological stress. These factors collectively impose a considerable burden on patients, healthcare systems, and society at large (Robinson, 2022). Peripheral nerve can spontaneously regenerate after neurotmesis if the damage is below a certain size, called limiting gap length (LGL). LGL depends on species and for humans is 3 cm. Long gaps exceeding the limiting size pose several obstacles to successful regeneration, impairing muscle functional recovery and the quality ofpatient’s life (Yannas and Hill, 2004). In such cases, strategies to promote nerve regeneration become crucial. Autografts (AGs) have long been the gold standard for nerve regeneration over the LGL, due to their compatibility and potential for promoting nerve regeneration (Ray and Mackinnon, 2010). However, despite their widespread use, AGs possess limitations. One significant drawback is donor site morbidity, where the harvesting of nerve tissue can lead to sensory deficits, scarring, and neuroma formation, thereby compromising the patient’s overall function and quality of life. Additionally, the availability of suitable donor nerves is limited, particularly in cases of extensive nerve injuries or multiple repairs, presenting challenges in achieving optimal nerve repair. Moreover, autografts do not precisely match the size and shape of the recipient nerve, leading to difficulties in achieving proper fiber alignment and functional recovery (Ray and Mackinnon, 2010). These limitations highlight the need for alternative approaches in nerve regeneration that can circumvent these challenges while promoting effective nerve repair and functional restoration. Various approaches, including tissue engineering, and the use of growth factors and cellular therapies, have been explored to address this challenge (Kemp et al., 2008; Kubiak et al., 2020). These approaches aim to create a supportive environment for axonal growth across the gap, facilitate Schwann’s cell (SCs) proliferation, stem cells differentiation and enhance neurotrophic support to promote nerve regeneration over longer distances. In the last years significant interest has risen for engineered nerve guidance conduits (NGCs) as alternative strategies for nerve repair. NGCs offer several advantages over autografts, including tunable physical and biochemical properties, controlled release of bioactive factors, and the potential for off-the-shelf availability. Interested readers could refer to (Deumens et al., 2010; Riva et al., 2024) for a thorough discussion on NGCs structural design, fabrication materials and strategies to enhance the nerve regeneration process. Few NGCs received EMA and FDA approval for the repair of short/mid gaps, such as Neuragen^®^, Neurolac^®^ and Reaxon^®^ made of type I collagen, poly-ε-caprolactone and chitosan respectively (Parker et al., 2021). However, NGCs are only approved for the reinnervation of mid-gap length, failing to restore muscle function for lesions over the LGL. Despite considerable preclinical research investigations, an effective strategy for significantly enhancing sensorimotor recovery following neurotmesis still remains elusive and further basic research effort is crucial to deepen our understanding of the biochemical and cellular mechanisms underlying nerve regeneration processes. A considerable amount of research, as detailed in (Navarro et al., 2007), has delineated SCs as mediators in peripheral nerve regeneration, supported by fibroblasts and immune cells that are recruited to the injury site following nerve damage. Following nerve injury, immune cells such as macrophages rapidly infiltrate the injury site. These cells are responsible for clearing debris, including myelin and axonal remnants, a process called Wallerian degeneration, which is essential for creating a favorable environment for regeneration. This involves the breakdown of the axon and myelin sheath, clearing the pathway for new axonal growth. Schwann cells play a pivotal role during this stage, dedifferentiating and proliferating to promote repair, while macrophages help remove debris. The subsequent regeneration process depends on the ability of the axon to extend and reconnect with its target tissue, which is guided by various cellular and molecular interactions. Over time macrophages switch from a pro-inflammatory (M1) to an anti-inflammatory (M2) phenotype, which is more conducive to tissue repair and regeneration (Dubový et al., 2013). Fibroblasts generate a structural framework termed the fibrin cable, which bridges the two severed nerve stumps. Fibroblasts also produce various components of the extracellular matrix, such as collagen, fibronectin, and laminin that provide structural support and biochemical cues that facilitate axonal regrowth and SCs migration and play a critical role in reconstructing the connective tissue architecture of the regenerating nerve (Gonzalez-Perez et al., 2013). Upon fibrin cable formation, SCs differentiate from their myelinating form to a pro-regenerative state, migrate in the interstump region and release neurotropic factors guiding the Bands of Büngner to support and guide regenerating axons that will subsequently reinnervate the target muscle (Fu and Gordon, 1997; Chan et al., 2014; Gordon, 2016b; Gordon, 2020).

Given the complex interplay among multiple cell types involved in nerve regeneration, it becomes evident that supporting peripheral nerves repair in limiting gap lesions requires the development of strategies capable of selectively modulate the aforementioned cellular pathways and eventually upregulating the ones with a pro-regenerative mechanism. These strategies also demand a high degree of precision and the development of systems capable of direct communication with target cells. Recent studies have highlighted the significant role of stimuli-responsive nanostructured materials in selectively modulating specific cellular functions involved in nerve regeneration processes. In response to external physical stimuli, these materials engage with target cells modulating their activity (Roy et al., 2010; Stuart et al., 2010). Conductive polymers and carbon-based nanomaterials can transmit external electrical signals to cells, thereby eliciting desired physiological responses (Zhang et al., 2018; Mariano et al., 2022). Furthermore, inorganic nanomaterials, such as metals or ceramics, can serve as nanotransducers, converting external stimuli (mechanical, electrical, optical, or magnetic) into other forms of energy (typically heat or electricity) to activate specific cellular functions in a wireless fashion (Li et al., 2021). The purpose of this Review is to provide a critical overview of the most relevant studies involving the use of nanostructured materials activated by external stimuli within the framework of peripheral nerve regeneration. The Review will also discuss their potential for enhancing the regeneration of limiting gap lesions and their clinical translation within engineered NGC designs as an alternative to autografts.

### 1.1 Cellular and molecular pathways in peripheral nerve regeneration

Peripheral nerve regeneration is regulated by several molecular pathways that coordinate cellular activities such as survival, proliferation, and axonal guidance (Allodi et al., 2012). These pathways include.

#### 1.1.1 The PI3K/Akt pathway

The PI3K/Akt (phosphoinositide 3-kinase/protein kinase B) pathway is one of the most important molecular pathways in nerve regeneration, especially for promoting cell survival and axon growth. Upon injury, various neurotrophic factors, such as nerve growth factor (NGF) and brain-derived neurotrophic factor (BDNF), bind to their respective receptors (such as Trk receptors) on neurons and Schwann cells, activating the PI3K/Akt signaling cascade. In this pathway, PI3K is activated and subsequently phosphorylates phosphatidylinositol-4,5-bisphosphate (PIP2) to generate phosphatidylinositol-3,4,5-triphosphate (PIP3). PIP3 then recruits and activates Akt, which promotes cell survival by inhibiting pro-apoptotic proteins such as BAD and activating pro-survival factors like mTOR (mammalian target of rapamycin). mTOR activation is critical for protein synthesis and axon elongation during nerve repair (Sun et al., 2017). In addition to its role in neuronal survival, Akt signaling supports SCs proliferation, migration, and the formation of Büngner bands, all of which are essential for directing axonal regeneration.

#### 1.1.2 MAPK/ERK pathway

The MAPK/ERK (mitogen-activated protein kinase/extracellular signal-regulated kinase) pathway is another essential signaling cascade in nerve regeneration, particularly for promoting cell differentiation, axon growth, and myelination. Neurotrophic factors such as NGF and BDNF also activate this pathway via Trk receptors. The activation of Trk receptors leads to the activation of Ras, a small GTPase, which in turn activates the MAPK/ERK cascade. This cascade involves a series of phosphorylation events that activate ERK. Once activated, ERK translocates to the nucleus and activates transcription factors such as CREB (cAMP response element-binding protein) and Elk-1, which promote the expression of genes involved in cell survival, growth, and differentiation. In neurons, MAPK/ERK signaling is crucial for axonal regrowth and guidance (Agthong et al., 2006). In SCs, this pathway supports differentiation and remyelination, key steps for restoring nerve function. The MAPK/ERK pathway also regulates the production of extracellular matrix (ECM) molecules, which are critical for providing structural support to the regenerating nerve.

#### 1.1.3 JAK/STAT pathway

The JAK/STAT (Janus kinase/signal transducer and activator of transcription) pathway is involved in both immune response and cellular proliferation during nerve regeneration. This pathway is activated by cytokines, such as interleukin-6 (IL-6) and leukemia inhibitory factor (LIF), which are secreted by macrophages and SCs in response to nerve injury. When cytokines bind to their receptors on the surface of SCs or neurons, JAK proteins are activated. These kinases phosphorylate the receptors, creating docking sites for STAT proteins, which are subsequently phosphorylated and dimerized. The activated STAT dimers translocate to the nucleus, where they initiate the transcription of genes involved in cell proliferation, survival, and immune regulation. In the context of nerve regeneration, the JAK/STAT pathway enhances SCs activation and the inflammatory response, which is necessary for clearing debris and preparing the environment for repair. This pathway also promotes axonal regrowth by modulating the expression of growth-associated genes (Lin et al., 2016).

#### 1.1.4 NGF, BDNF, and GDNF signaling

Neurotrophic factors such as NGF, BDNF, and glial cell line-derived neurotrophic factor (GDNF) are critical for supporting nerve regeneration by activating several signaling pathways, including PI3K/Akt and MAPK/ERK. These factors bind to their respective receptors—TrkA for NGF, TrkB for BDNF, and Ret for GDNF—triggering downstream signaling cascades that promote neuronal survival, axonal growth, and synaptic plasticity (Terenghi, 1999). NGF plays a vital role in sensory neuron survival and the extension of axons, while BDNF is particularly important for motor neurons and synaptic formation. GDNF supports the survival and regeneration of motor neurons and also enhances SCs migration and the production of neurotrophic factors. These neurotrophic factors create a neuroprotective environment by activating intracellular signaling pathways that reduce apoptosis, promote protein synthesis, and stimulate axonal outgrowth. They also interact with other signaling molecules, amplifying the regenerative response.

#### 1.1.5 Calcium signaling pathway

2 Ca^2+^ ions serve as important second messengers in many cellular processes, including nerve regeneration. Changes in intracellular calcium levels regulate the activity of growth cones at the tip of regenerating axons, influencing axon guidance and directionality. Calcium influx through voltage-gated calcium channels or mechanosensitive channels can activate several downstream effectors, including calmodulin and calcineurin, which modulate cytoskeletal dynamics and growth cone motility (Berridge, 1998). In SCs, calcium signaling is essential for migration and myelination. Calcium influx also promotes the release of neurotrophic factors and ECM components, further supporting axon regeneration.

## 2 Physical stimuli to modulate cellular functions in nerve regeneration

**FIGURE 1:**
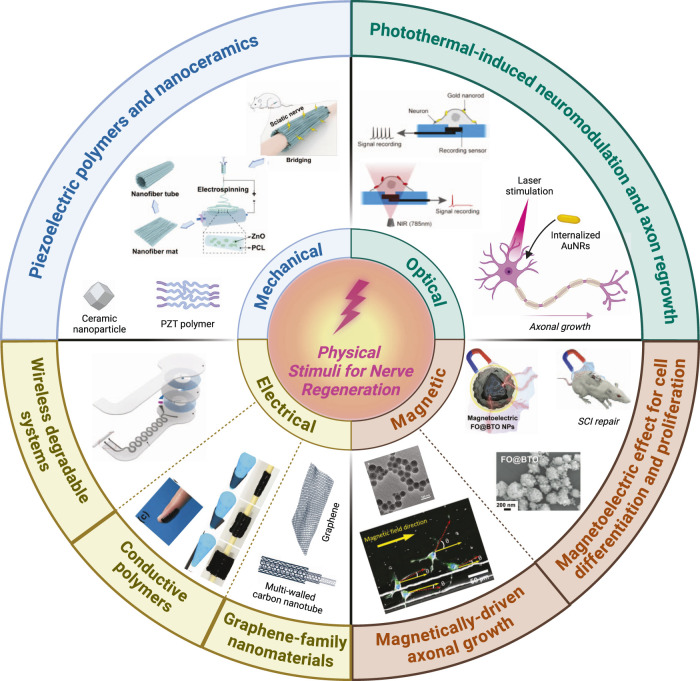
Summary of the most widely used physical stimuli to enhance peripheral nerve regeneration process. Schematic representation of piezoelectric polymers and nanoceramics adapted and reproduced with permission from (Mao et al., 2022). Photothermal-induced neuromodulation and axonal regrowth adapted and reproduced with permission from (Yoo et al., 2014). Wireless degradable systems adapted and reproduced with permission from (Koo et al., 2018). Conductive polymers adapted and reproduced with permission from (Liu et al., 2021). Magnetically-driven axonal growth adapted and reproduced with permission from (Kim et al., 2011). Magnetoelectric effect for cell differentiation and proliferation adapted and reproduced with permission from (Zhang et al., 2021). Created with Biorender.com.

The successful regeneration of peripheral nerves following injury relies on the coordinated response of various cellular components, such as SCs, and supporting cells. External physical stimuli, ranging from mechanical forces to electrical field and magnetic field, are emerging as powerful and promising tools to modulate cellular functions and promote nerve regeneration (Figure 1).

The subsequent section will examine the most promising research on the application of physical stimuli to facilitate peripheral nerve regeneration. These studies will be organized according to the type of physical stimulus employed. Initially, the focus will be on investigations related to electrical stimulation (ES), which represents the earliest approach to employing physical stimuli for nerve regeneration. This stimulation is applied through traditional electrodes or via nanostructured materials, such as conductive polymers and graphene-based compounds. Additionally, the focus will extend to other physical stimuli that have shown particularly promising results, given their potential to wirelessly activate nanostructured materials functioning as nanotransducers to modulate specific cellular activities. In particular, wireless mechanic, optical and magnetic stimulation will be discussed, as stimuli to induce energy conversion through nanostructured materials used as nanotransducers to modulate cellular functions to support peripheral nerve regeneration.

### 2.1 Electrical stimulation to promote nerve regeneration

Historically, ES has been thoroughly studied as possible mechanism to enhance nerve regeneration *in vivo* (Gordon, 2016a; 2020; Gordon and English, 2016). The biochemical basis on how ES can facilitate nerve regeneration process has been thoroughly studied. Shifts in transmembrane potential can alter cell proliferation and trigger self-propagation of action potential along the axon (Haastert-Talini et al., 2011). In the neuronal cell body, electrical signals cause an over-expression of regenerative-associated genes, enhancing the production of neurotrophic factors such as brain-derived neurotrophic factor (BDNF) and its receptor TrkB. Through a calcium dependent mechanism, the upregulated BDNF and TrkB increase the upregulation of regenerative associated genes (RAGs) such as Tα-1 tubulin and growth associative protein-43 (GAP-43) via the cyclic adenosine monophosphate (cAMP) pathway (Bisby and Tetzlaff, 1992; Gordon et al., 2009). After electrical stimulation SCs release neurotrophic factors through a calcium dependent mechanism. The ES-induced BDNF release required calcium influx through T-type calcium voltage-gated calcium channel (VGCC) and calcium mobilization from internal stores. In addition, activation of CREB-mediated gene expression was also involved in the ES-induced BDNF release (Haastert-Talini et al., 2011; Willand et al., 2016; Zuo et al., 2020a).

ES can traditionally be generated by electrodes implanted to the proximal nerve stump after a neurotmesis. Gordon and colleagues demonstrated that ES promotes and accelerates nerve regeneration (Brushart et al., 2005; Gordon et al., 2008). ES was performed on rodent femoral nerves at 20 Hz with 3–5V for various time duration (from1 hour to 2 weeks). Their results showed that a single session of ES immediately applied 1 h following AG implantation enhances axon regeneration, in a 10 mm nerve autograft (Zuo et al., 2020b). ES could augment cAMP levels in neurons and accelerate the upregulation of neurotrophic factors and their receptors within neurons and SCs. Following these encouraging results, human trials were attempted. In a randomized double-blind study, patients with complete transection of a digital nerve underwent nerve repair and received either 1 h of 20 Hz ES or sham stimulation postoperatively (Wong et al., 2015). With a nerve regeneration distance of 2–6 cm, the group receiving 20 Hz ES showed significantly improved sensory outcomes, achieving complete recovery to baseline levels by 5–6 months, compared to the sham group. Moreover, chronic ES was also studied by Navarro and colleagues with an attempt to investigate whether periodic ES (1h daily for 4 weeks) could further improve reinnervation (Asensio-Pinilla et al., 2009). However, no significative changes were evidenced in animal treated with chronic ES, respect to those treated with a single ES session after injury, suggesting that the adjuvant effect of this type of ES may occur in the early stages of the nerve regeneration process. Traditional ES protocol poses some limitation in the clinical practice, as it increases operative time and creates additional complexity for both the surgeon and hospital. Furthermore, the use of non-portable electrical stimulator and external wires that can cause chronic inflammation process and nerve damage complicate the systematic and safe utilization of this approach. Materials science and novel biomaterials offer potential for biocompatible, bioresorbable implanted nerve stimulators, reducing the need for intraoperative stimulation. A recent study introduced a wireless, bioresorbable nerve stimulator with cuff electrodes, showing that multiple 1-h daily sessions of monophasic electrical impulses for 6 weeks days significantly accelerated muscle reinnervation compared to sham stimulation (Koo et al., 2018). This approach demonstrates that the use of a device characterized by more sophisticated materials, capable of delivering electrical stimulation deeper into the treated nerve, may ensure good regenerative performance even for long-term applications. For this reason, efforts in the use of biomimetic and nanostructured materials, capable of ensuring an even closer integration with the tissue to be treated, are yielding promising results in terms of enhancing the efficiency of the nerve regeneration process.

### 2.2 Electroactive organic materials

Researchers have been developing or utilizing various electroactive organic materials, including conductive polymers and graphene family materials, to boost peripheral nerve reconstruction by enabling the local delivery of electrical stimulus (Zarrintaj et al., 2020).

#### 2.2.1 Conductive polymers

The softness of conductive polymers renders them mechanically more compatible with cells and tissues in comparison to metals and inorganic materials. The most popular conductive polymers implemented in neural tissue engineering are polyaniline (PANI), polypyrrole (PPy), poly (3,4-ethylene dioxythiophene) (PEDOT), and polythiophenes (PT) owing to their facile synthesis and functionalization, tunable properties, and biocompatibility. For instance, Dong et al. have developed a near-infrared (NIR) light-stimuli responsive and stretchable conductive hydrogel (CPH) through the copolymerization of polyacrylamide (PAM) and PANI (Dong et al., 2020). The conductivity of PAM/PANI hydrogel was enhanced upon NIR light exposure, which could increase the conduction of bioelectrical signal. *In vivo* studies in the rat sciatic nerve model with a 10 mm-long lesion confirmed the regenerative effect of CPH, although autograft control as a comparison was not performed. Other examples of PANI in neuroregeneration have been reported only *in vitro* (Shrestha et al., 2019; Kim et al., 2020).

**FIGURE 2:**
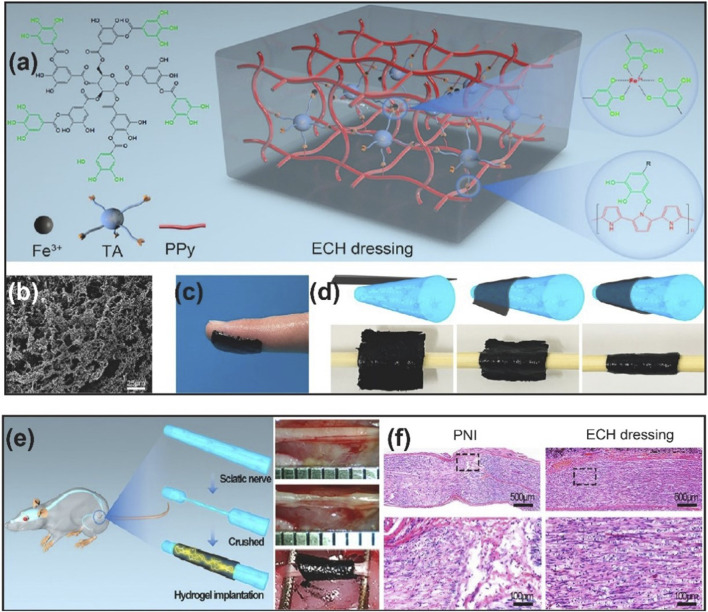
**(A)** Schematic representation of ECH dressing and its chemical structure **(B)** SEM image of ECH **(C)** Adhesive properties of ECH **(D)** Self-curling properties of ECH **(E)** ECH implantation in diabetic rat with crushed sciatic nerve **(F)** H&E staining of the sciatic nerve on the 28th day after ECH implantation. Reproduced with permission from (Liu et al., 2021). Copyright Elsevier.

PPy has been the most widely explored polymer for developing conductive NGCs. Among the other conductive polymers for developing NGCs in the recent literature, PPy-based NGCs are the ones presenting more *in vivo* studies. In a recent study, conductive composite NGC has been reported (Zhao et al., 2020). The exterior wall of NGC was based on electrospun silk fibroin (SF) fibers, whereas the interior wall was decorated with 3D-printed PPy/SF composite fibers. As-prepared NGC showed good biocompatibility and stability under ES. These scaffolds increased Schwann cell viability, proliferation, migration, and upregulated gene expression of neurotrophic factors (NGF, BDNF, NT-4/5) upon ES. Besides, a 10 mm rat sciatic nerve defect was repaired under ES evaluated by axonal regeneration and myelination. The authors further investigated the functional recovery as well as the molecular pathway of observed enhanced regeneration. They concluded that when ES was applied, PPy/SF composite NGC enabled the expression of growth factors and activated the mitogen-activated protein kinase (MAPK) signal transduction pathway, which regulates essential cytological processes depending on the stimulation and cell type. Another example of the utilization of the electroactive property of PPy for neural regenerative medicine applications has been demonstrated (Liu et al., 2021). An electroconductive hydrogel (ECH) based on PPy, tannic acid (TA), and Fe^3+^ has been fabricated (Figures 2A, B). ECH exhibited good adhesive and self-curling properties such that it could be wrapped around the injured nerve, as shown in Figures 2C, D. ECH provided a stable electrical connection to electrogenic nerve tissues. As indicated by the *in vitro* experiments, ECH promoted Schwann cell migration and adhesion. Through the MEK/ERK pathway, the ECH enhanced axonal regeneration both *in vitro* and *in vivo*. Muscle atrophy was prevented while preserving the function after crushed sciatic nerve injury in diabetic rats (Figures 2E, F).

As mentioned in the previous examples, various materials have been blended with PPy to obtain conductive NGCs. Jing and coworkers developed a degradable conductive NGC made of PPy and PLGA composite (Jing et al., 2018). They also prepared fibers through the deposition of PPy onto electrospun parallel PLGA fibers and used these fibers as filling material inside the NGC. The resulting conductive NGC was found to effectively repair sciatic nerve injury in rat models owning comparable performance to autograft groups. The authors attributed the efficient regenerative effect to the synergistic effect of conductivity and contact guidance provided by aligned fibers. Song and coworkers have recently taken a major step forward in the regenerative rehabilitation approach by improving the therapeutic potential of human stem-cell-based therapies through ES (Song et al., 2021). They have fabricated NGC made of PPy. Human neural progenitor cells (hNPCs) were seeded onto PPy-based NGC and underwent ES. Sensory and motor functions in the rat sciatic nerve injury model were improved more significantly after ES applied hNPCs-containing PPy-based NGC compared to either treatment alone in the first 2 weeks of implantation. It was found that ES had long-term effects on nerve regeneration and distal nerve reinnervation by increasing the expression of tyrosine kinase receptors (Trk) receptors, well known for binding to neurotrophic factors. Another blend of PPy has been prepared by Jiao and colleagues (Jiao et al., 2021). In their study, they have constructed size-tunable microfluidic hollow fibers with enhanced stiffness and elasticity using a conductive triple network consisting of PPy, sodium alginate/Ca^2+^, and polyacrylamide (PA). The conductivity of the as-prepared HF has been 0.32 S/m higher than the native sciatic nerve. Instead of ES, a pulsed magnetic field (MF) has been applied. It has been shown that pulsed MF, which generates electromotive forces non-invasively on the hollow fibers and promotes migration, proliferation, and NGF secretion of Schwann cells, was more effective than ES therapy since the electrodes outside the body were more likely to cause complications. Additionally, the exogenous NGF-7S was loaded into hollow fibers and found to be released slowly from the hollow fibers, which promotes neural differentiation of PC12 cells. In light of findings from *in vitro* experiments, pulsed MF and NGF-7S were combined and implemented in rats with a 5 mm lesion of sciatic nerve injury. This system could afford successful nerve regeneration and functional recovery *in vivo*, although it could not surpass the autograft performance. Also, it should be noted that the 5 mm-lesion model is way below the LGL.

PEDOT and its derivatives have also drawn attention as conductive polymers not only for peripheral nerve regeneration but also for a wide range of neural applications (Dimov et al., 2022). For neural tissue engineering, Babaie and colleagues have produced various compositions of electrospun conductive polyvinyl alcohol (PVA)/PEDOT scaffolds (Babaie et al., 2020). When only 1 wt% of PEDOT was blended with PVA, topographical features and electrical conductivity of fibrous scaffolds were enhanced. These scaffolds improved the metabolic activity of rat mesenchymal stem cells (MSCs), and upon ES, the differentiation of MSCs into neuron-like cells was achieved. Besides, scaffold samples stimulated with electrical current expressed the nestin gene, a gene expressed in nerve cells where they are involved in the radial growth of axons, 1.5 times more significantly than scaffold samples without ES.

#### 2.2.2 Graphene family materials

The discovery of graphene in 2004 has been one of the most significant scientific breakthroughs (Novoselov et al., 2004). It has initiated the development of a whole new class of nanomaterials exhibiting unique physical, chemical, and biological properties (Ghosal et al., 2021). GFNs include graphene derivatives such as monolayer graphene, multilayer graphene, carbon nanotubes (CNTs) (single-walled CNTs or multi-walled CNTs), buckyball, graphite, graphene oxide (GO), reduced graphene oxide (rGO) and graphene quantum dots (GQDs). GFNs have a large surface area and therefore are able to interact with biomolecules (Sanchez et al., 2012).

In the context of peripheral nerve regeneration, it was shown that stem cells could be differentiated into nerve cells by GNFs. Additionally, GNFs can strengthen functional neuron networks (Lee et al., 2018). From the overview of recent literature on electroactive organic materials, GNFs have taken a more widespread place in state-of-the-art peripheral nerve injury treatments compared to conductive polymers. However, both classes suffer from limited animal studies. Recently, CNT-poly (ethylene glycol) (PEG) hydrogel composites have been presented (Ye et al., 2021). An *in-situ* polymerization of PEG around a preformed meshwork of CNTs resulted in CNT-PEG hydrogel composites with high electrical conductivity. The authors have reported elaborate *in vitro* studies on composites. Firstly, the CNT-PEG hydrogel composite has been shown to provide long-term survival as well as differentiation for PC-12 cells seeded onto it. Secondly, a higher ratio of neurons to astrocytes and greater synaptic connectivity has been observed in adult neural stem cells (NSCs) cultured on the composites. Moreover, primary hippocampal neurons cultured on composites demonstrated that these neurons maintained morphological synaptic features, as well as spontaneous calcium oscillations, which were found to be comparable to cells cultured under control conditions. According to these results, neuronal differentiation could indeed be enabled while maintaining neuronal homeostasis by the composites. Similarly, another CNT-based hydrogel composite has been reported (Hu et al., 2022). The composite was fabricated through the combination of methacrylated gelatin hydrogel (GelMA) and super-aligned CNT sheets (SACNTs) utilizing the biocompatibility of GelMA and the electrical conductivity of CNTs. GeLMA-SACNT composites facilitated spiral ganglion neurons’ regeneration and directional regrowth (SGNs).

**FIGURE 3:**
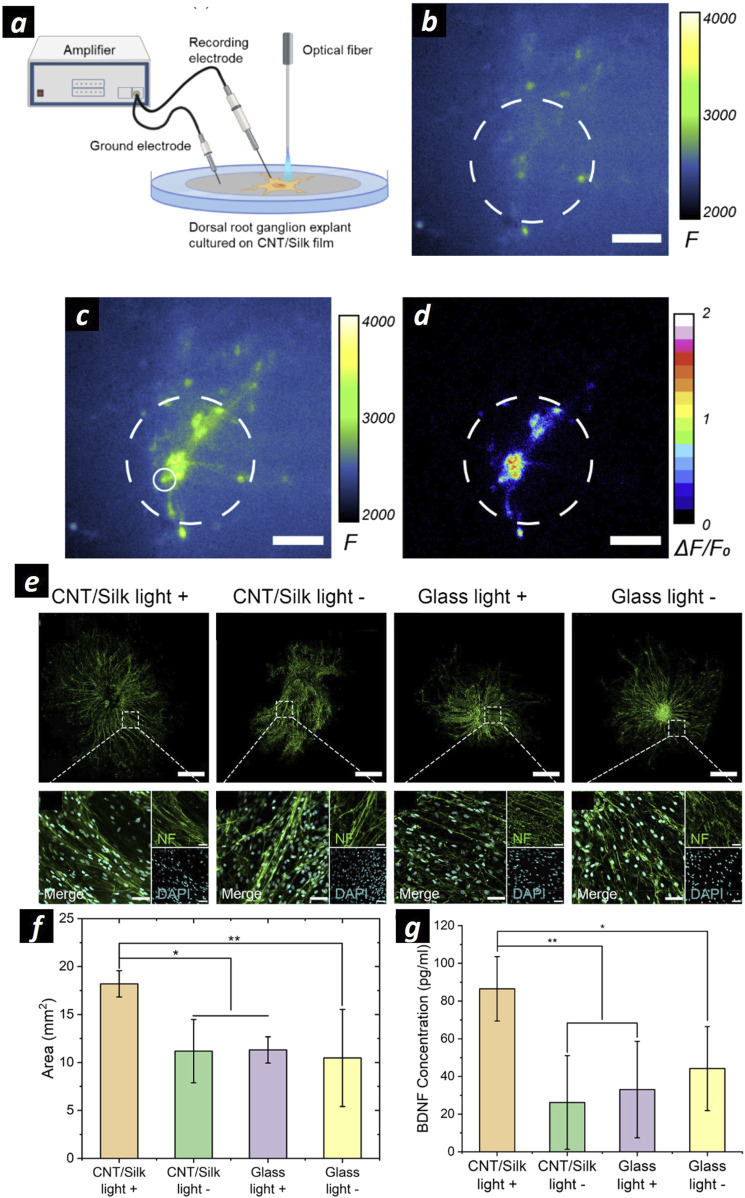
**(A)** Schematization of the experimental setup for extracellular recording and PA stimulation on DRG cells cultured onto CNT/silk films. **(B, C)** Representative calcium images of neuronal cells at DIV 14 cultured on CNT/silk film before **(B)** and after **(C)** PA stimulation. **(D)** Map of the maximum fluorescence change ΔF/F0 induced by the PA stimulation. Dashed lines: Illumination area. Scale bars: 100 µm. **(E)** Representative confocal microscopies of DRG cells stained for neurofilament (green): DRGs cultured on CNT/silk film with laser illumination (CNT/silk light +), and without laser illumination (CNT/silk light −). DRG cells cultured on a glass bottom dish with laser illumination (glass light +) and without laser illumination (glass light −). Scale bar: 1 mm. In the inset high-resolution confocal images of DRG cells stained with Anti-Neurofilament 200 (NF) for neurites (green) and DAPI for nuclei (cyan). Scale bar: 50 µm. **(F)** Average neurite coverage area for DRGs in four groups. **(G)** Average concentrations of BDNF of PA-stimulated and unstimulated DRGs. Samples were collected 24 h after PA stimulations. PA stimulations were provided every 2 min within the total duration of 1 h. The stimulation conditions were a laser train duration of 5 ms, 1.7 kHz repetition rate, and a pulse energy of 14.7 µJ. Adapted and reproduced with perission of (Zheng et al., 2022). Copyright ACS Publications.

Encouraged by the reported *in vitro* results, CNTs are promising materials for regenerative medicine of peripheral nerves, and their effect is worth investigating *in vivo* models to improve their clinical translation. For this purpose, CNTs should be devised in NGC form. Zhou and colleagues developed a conductive NGC based on CNT-embedded polycaprolactone fumarate (PCLF) by using UV-induced crosslinking (Zhou et al., 2018). Comparing the CNT-integrated PCLF NGC to the PCLF NGC, the PCLF-CNT NGC exhibited improved surface roughness, lower polymer impedance, a lower tensile modulus, and faster biodegradability. However, they showed the regenerative ability of NGC under ES only *in vitro*. Recently, GFNs have been employed to harness a promising diagnostic technique called Photoacoustic Imaging (PAI). PAI is an emerging technology that leverages laser-generated ultrasound. It involves using pulsed laser light to excite absorbing molecules, causing them to undergo thermoelastic expansion and release acoustic waves. This process enables high-resolution imaging of deeper tissues (L. Deán-Ben et al., 2017; Li et al., 2020). Polyethylene glycol (PEG)-functionalized carbon nanotubes (CNTs), recognized for their high photoacoustic efficacy, are embedded within silk/fibroin to develop biocompatible and flexible photoacoustic materials (Zheng et al., 2022). This study demonstrates that these photoacoustic-functionalized scaffolds facilitate non-genetic neuronal activation with spatial precision defined by the light illumination area, thereby enhancing neuronal regeneration (Figures 3A–D). Specifically, photoacoustic stimulation using a Q-switched 1,030 nm ns laser, operating at a repetition rate of 1.7 kHz and a pulse width of 3 ns, resulted in a 1.74-fold increase in neurite outgrowth in a rat dorsal root ganglion model compared to the unstimulated controls (Figures 3E, F). Additionally, the study confirmed that the increased neurite outgrowth associated with photoacoustic stimulation is correlated with upregulated expression of the neurotrophic factor BDNF (Figure 3G).

Apart from CNTs, other GFNs have also been exploited. Qian et al. developed multi-layered porous NGC using 3D printing and layer-by-layer casting (Qian et al., 2018). The NGC was based on polycaprolactone (PCL) and single- or multi-layered graphene and coated with polydopamine (PDA) and arginylglycylaspartic acid (RGD) to improve cell adhesion. Another example of graphene-based NGC for *in vivo* nerve regeneration has been recently reported (Huang et al., 2021). An engineered double network (DN) hydrogel scaffold has been synthesized with graphene mesh as support and loaded with netrin-1. An entangled hydrogel that was obtained via rapid exchange of ions and ultraviolet irradiation was composed of alginate and gelatin-methacryloyl, which provides mechanical strength and outstanding biocompatibility. In the meantime, graphene mesh induces Schwann cell proliferation and facilitates their alignment. Furthermore, netrin-1 promoted SCs migration via the phosphorylation of p38, MAPK and Akt. The DN hydrogel NGC has good electrical conductivity. A 100 ng/mL concentration of netrin-1 also directs axon pathfinding and neuronal migration. The netrin-1-loaded graphene mesh tube/DN hydrogel nerve scaffold was shown to be superior to autografts in promoting peripheral nerve regeneration and muscle restoration in a rat sciatic nerve model with a 10 mm lesion. Additionally, the scaffold + netrin-1 groups have a significantly higher expression of vWF (a protein marker of endothelial cells) respect to control, and a higher level of CD31, which correlates with good intraneural angiogenesis.

Overall, GFNs lead to high interactions between neural cells and conductive functions, therefore, were considered promising materials to address peripheral nerve injuries. On the other hand, there are shortcomings associated with their clinical translation (Aleemardani et al., 2022). Since GFNs are often classified as hazardous materials, extensive safety assessments are a significant drawback preventing further clinical studies with GFNs (Bianco, 2013). GFNs accumulation could cause a high generation of intracellular and extracellular reactive oxygen species (ROS). Most of the reported research on GFNs remains at *in vitro* stage. The major limitation of the current studies lies in the simplicity of the cytotoxicity analyses used to assess scaffold toxicity. Despite the results being acceptable, they are not sufficient for clinical implementation; hence, they should be validated by thorough evaluations. Furthermore, long-term *in vivo* toxicity and biocompatibility studies should be carried out as there is currently quite limited literature available on carbon-based material.

### 2.3 Nanostructured ceramic and metallic materials as wireless nanotransductors

Scaling down materials dimensions at the nanoscale (1–100 nm) allows for the exploration of extraordinary physical properties not exhibited at the macro- or micro-scale, such as superparamagnetism and localized surface plasmon resonance (LSPR) (Di Ventra et al., 2004). At the nanoscale, matter’s properties cease to depend on its mass, and even little changes in the material’s geometry and shape can have a drastic effect on quantum states and, therefore on the material properties. In the last decades, metallic and ceramic nano-objects that could convert external energy into another were investigated as nano-transducers for neuromodulation purposes (Li et al., 2021). In this regard, the adsorbed magnetic and light sources, as well as mechanical stresses affecting the nanostructure, are converted into an electrical field or local temperature increase that can modulate specific biochemical pathways by interacting with the cell surface.

#### 2.3.1 Piezoelectric nanomaterials

Specific interest has grown around piezoelectric nanostructures, as recent *in vitro* studies evidenced the possibility of modulating bone and cartilage cells orientation, arrangement, and proliferation thanks to mechano-electrical transduction that alter substrate’s surface charge and induce intracellular Ca^2+^ influx that triggers gene transcription associated to proliferation (Khare et al., 2020; Shuai et al., 2020). Moreover, piezoelectric stimulation has been demonstrated to induce cell differentiation, as reported in a recent *in vitro* investigation with mesenchymal stem cells (Damaraju et al., 2017). In the field of Neuroscience, *in vitro* investigation showed that piezoelectric NMs induced neurite length of PC12 cells and electrically stimulated SH-SY5Y neuroblastoma cells using boron nitride nanotubes (BNNTs) and tetragonal barium titanate nanoparticles (BTNPs) respectively (Ciofani et al., 2010; Marino et al., 2015). In these studies, nanoparticles were incubated with cells and ultrasound (US) was used to impart mechanical stresses to NPs and convey electrical stimuli to the cells. Interestingly, US-stimulated PC12 cells upon BNNTs internalization exhibited 30% longer neurite than the control culture, and BNNTs showed good cytocompatibility towards neuronal cells. The Ca^2+^ influx generated by US stimulation and the NGF-specific receptor TrkA were demonstrated to play a role in PC12 neurite extension. Moreover, US stimulation of SH-SY5Y neuroblastoma cells previously incubated with BTNPs triggered action potential and elicited a cellular response in terms of Ca^2+^ influx into the cytoplasm. The authors claimed that the localization of NPs at the plasma membrane and their subsequent polarization upon US stimulation was able to open voltage-gated Ca^2+^ channels, thus generating high-amplitude calcium transient respect to the non-stimulated control culture. These data proved that local dipole fluctuations and the calcium waves generated by voltage-gated channels activation and variation of cell membrane capacitance due to US NMs stimulation at the neuronal cell membrane interface could effectively generate an action potential and enhance neurite outgrowth. Moreover, activation of cAMP, MEK and MKK pathways upon voltage-gated channel activation can upregulate cellular functions associated with neural regeneration, such as cell proliferation and myelination maturation (Qian et al., 2021a). Electrical stimulation of intracellular cAMP initiates the increase, and downstream of cAMP, protein kinase A encourages the expression of genes responsible for nerve regeneration, facilitating axonal elongation. Further research in this field pointed out promising *in vivo* investigation with the attempt to provide the piezoelectric potential to NGCs design. Recently Mao and colleagues presented an electrospun ZnO-loaded PCL nanofibers rolled to create an NGC to reconnect a 10-mm gap of the sciatic nerve of the rat (Mao et al., 2022). *In vitro* test on rat’s SCs performed using Flexcell tension system to trigger piezoelectric effect showed a increase of NGF and VEGF production respect to the non-stimulated control, evidencing a role of piezoelectric effect in enhancing neurotrophins production by SCs. mRNA sequencing analysis identified that genes expressed during piezoelectric stimulation were significantly enriched in several pathways, including the PIK3-Akt, MAPK, and RAS signaling pathways, which are closely associated with the RET signaling pathway. Further gene network analysis revealed that several upregulated genes were associated with GRB2, which was demonstrated to induce nerve regeneration after piezoelectric stimulation. *In vivo* tests showed enhanced nerve regeneration of ZnO-loaded PCL NGC respect to neat PCL NGCs, as reported by TEM images and Luxol Fast blue staining. Further evidence of enhanced nerve regeneration through piezoelectric effect was provided by evaluation of sciatic function index SFI and higher β-actin, S100 and NGF levels shown by Western blotting analysis and immunohistochemistry.

It is worth to note that to generate appropriate electrical stimuli to induce a cellular response, proximity between piezoelectric materials and cells is required. In an interesting recent study, piezoelectric ZnO nanoparticles were added to the inner layer of an NGC to ensure greater contact with cells. The group of Cunyi Fan published two recent works presenting two types of piezoelectric PCL-based NGCs: one loaded with ZnO NPs (Qian et al., 2020) (Figure 5_1a) and the other one loaded with BNNTs nanosheets (Qian et al., 2021b). In both studies, three groups (piezoelectric NGC, PCL NGC, and autograft) were employed to bridge a 15-mm nerve defect in the rat sciatic nerve. The animals were allocated to two subgroups: the treadmill running group and the non-treadmill running group. The physical exercise was used to deform the piezoelectric NGC to generate electrical stimuli for regenerating axons: rats received a 30 min running every day at 3 m/min initially and was increased 2 m/min every 2 weeks. Piezoelectric NGCs’ regenerative performance was found superior to PCL counterpart and resembles the autograft as shown in Figure 5_1b and 5_1c, both in terms of nerve morphology and muscle functional recovery. Moreover, immunohistochemical analysis showed increased protein expression levels (S100, Tuj1, NF160, and CD34) in the treadmill running group with respect to the non-treadmill running group. In particular, myelin basic protein (MBP), β-III-tubulin (Tuj1) and glial fibrillary acidic protein (GFAP) overexpression thanks to piezoelectric effect resulted in improved nerve regeneration in the treadmill running group with respect to the non-treadmill running group. Piezoelectric effect was also used as stimulus to trigger electrical stimulation in a silk/fibroin multichannel NGC functionalized with PEDOT:PSS conductive polymer (Ma et al., 2023). A combined poly (L-lactic acid-co-caprolactone) (PLCL) and polyvinylidene fluoride (PVDF) was created to construct a PVDF/PLCL composite film with piezoelectric properties. Upon implantation to repair 10 mm gap of the sciatic nerve of the rat, animal movement triggered piezoelectric effect which was then converted into electrical stimulation thanks to the PEDOT:PSS component within the NGC. This effect induced maturation and myelination of SCs *in vitro*, confirmed by Western blot and RT-qPCR, which revealed transcription of genes related to myelination, including neuronal cellular adhesion molecules (NCAM), nerve growth factor (NGF) peripheral myelin protein 22 (PMP22) and MBP. Support to nerve regeneration process was confirmed *in vivo*, with immunohistochemistry confirming remarkable regenerated nerve morphology with no appreciable differences with respect to autograft. However, autograft was still more efficient in restoring motor function, as demonstrated by compound muscular action potential (CMAP) and nerve conduction velocity (NCV) analysis.

**FIGURE 4:**
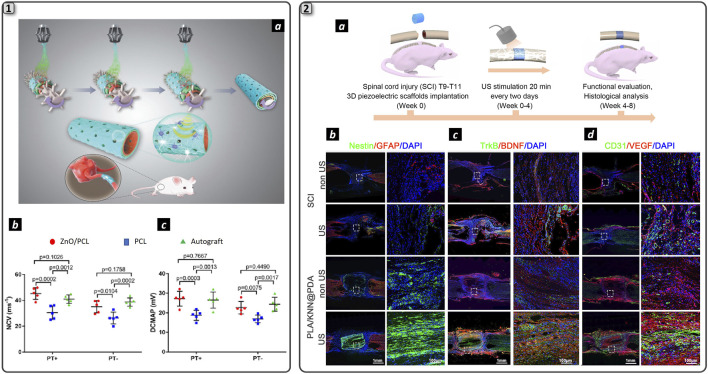
Piezoelectric nanomaterials for neural regeneration. 1) Schematization of the fabrication process and function of ZnO/PCL NGC (a). Electrophysiology measurements displaying nerve conduction velocity (b) and distal compound motor action potential (c) for the experimental groups. Adapted and reproduced with permission of (Qian et al., 2020). Copyright Wiley & Sons. 2) Schematization of spinal cord injury repair employing US-driven wireless ES (a). Immunofluorescence double staining of Nestin (red) and GFAP (green) (b), of TrkB (red) and BDNF (green) proteins (c) and of CD31 (red) and VEGF (green) proteins in the two experimental groups: spinal cord injury (SCI) and hydrogel (PLA/KNN@PDA) treated with and without US stimulation. DAPI marked cell nuclei. Scale bars are 1 mm and 100 µm in the large-scale images and insets respectively. Adapted and reproduced with permission of (Chen et al., 2022). Copyright ACS Publications.

**FIGURE 5:**
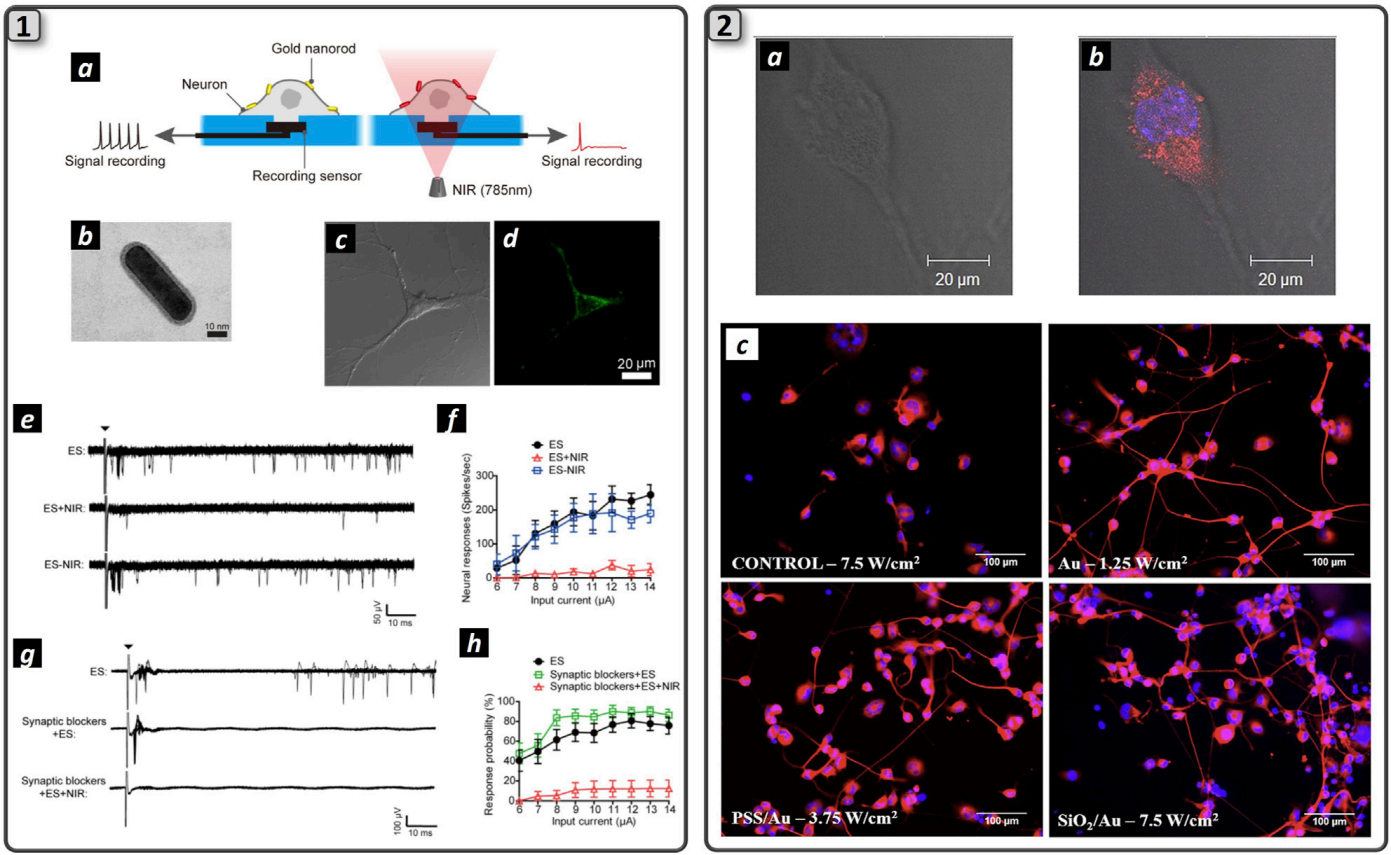
Plasmonic nanomaterials trigger action potential in neuronal cells. **(1)** The schematization of the process of NIR laser-assisted axon potential blockage enabled by NIR-resonant Au NPs (a). TEM image of NH2-PEG-coated gold nanorod (b). Bright-field (c) and fluorescence (d) images of neurons treated with fluorescently labeled NH2-PEG-gold nanorods. MEA recordings following the electrical stimulation with or without NIR irradiations (e) Quantification of spike rates of GNR-treated neurons upon NIR irradiation simultaneously with repeated electrical stimulations at different input current levels (f) MEA recordings of electrically evoked GNR-treated neurons upon NIR irradiation after synaptic blockade (g). Quantification of neural responses with synaptic blockers (h). Adapted and reproduced with permission of (Yoo et al., 2014). Copyright ACS Publications. **(2)** Merging of bright field image (a) and fluorescence microscopy (b) of an NG108-15 neuronal cell cultured with RhB labeled AuNRs. Cells were cultured for 5 days in serum-free medium and stained for DAPI (blue). RhB-labeled NRs are imaged in red. Clusters of RhB-labeled AuNRs uniformly dispersed inside the cytoplasm and excluded by cell nucleus. Fluorescence microscopies of NG108-15 neuronal cells cultured with Au, PSS/Au, and SiO_2_/Au NRs and labeled with b-III tubulin (red) and DAPI (blue). Neuronal cells were irradiated with the 780 nm laser after 1 day of culture in serum-free medium and then immunolabeled after a further 3 days of culture. The irradiance used for laser exposure is indicated in the figure (c). Adapted and reproduced with permission from (Paviolo et al., 2013). Copyright Wiley & Sons.

These results evidenced that electrical stimuli delivered by the piezoelectric effect could have an impact in enhancing nerve regeneration, and the authors postulated that this effect is due to SCs responsivity to electrical stimuli that trigger cellular function as evidenced with *in vitro* preliminary analysis. However, this approach presents a significative limitation since it is already known in the literature that treadmill exercise itself promotes nerve regeneration, causing an increase in the production of neurotrophic factors by cells (Asensio-Pinilla et al., 2009). Furthermore, using physical movement as a method to stimulate the NGC and induce the piezoelectric effect cannot result in a spatiotemporally repetitive electrical stimulation, posing challenges in standardizing the piezoelectric stimulation protocol. For this reason, further characterization will be needed to assess the real contribution of the piezoelectric effect to nerve regeneration compared to the effect of physical exercise alone. In order to enhance the repeatability and safety of piezoelectric stimulation, a relevant study was recently published in the framework of spinal cord injury (Chen et al., 2022) (Figure 5_2). Here, a 3D scaffold made by electrospun PLA nanofibers incorporating a biodegradable potassium sodium niobate (K0.5Na0.5NbO3, KNN) and polydopamine (PDA (named PLA/KNN@PDA) provided US-drive wireless ES to restore spinal cord defects (Figure 5_2a). Preliminary *in vitro* analysis reported successful differentiation of neural stem cells (NSCs) upon PZT stimulation and *in vivo* experiments showed the potential of the 3D scaffold to restore a 2-mm spinal cord injury with 4-week US treatments, respect to the non US-stimulated control). Particularly, immunofluorescence costaining of the intermediate filament protein Nestin (green) and glial fibrillary acidic protein (GFAP, red) was conducted to assess endogenous neural differentiation at the injury site after 8 weeks of repair (Figure 4_2b). The PLA/KNN@PDA-US group exhibited the highest relative Nestin intensity among all groups. TrkB/BDNF costaining in longitudinal spinal cord sections (Figure 4_2c) revealed that the expression levels of TrkB and BDNF in the PLA/KNN@PDA-US and PLA/KNN@PDA groups were significantly elevated compared to the SCI and SCI-US groups. Finally, a significant increase in vascular endothelial cells labeled with platelet endothelial cell adhesion molecule-1 (CD31) and vascular endothelial growth factor (VEGF) in the PLA/KNN@PDA-US group compared to the other control groups (Figure 4_2d). This suggests enhanced angiogenesis in the PLA/KNN@PDA-US group, supporting improved tissue repair and recovery. This recent study has uncovered a promising avenue in the context of neural regenerative medicine: the utilization of a wireless approach to deliver ES therapy. This method harnesses ceramic nanomaterials as nanotransducers, converting external US stimuli into microcurrents precisely transported at the cellular level. This process is facilitated by a 3D architecture of the regenerative scaffold, thanks to the use of biomaterials that support cellular proliferation. Another relevant study (Xu et al., 2024) employs electrospun piezoelectric polymer nanofibers doped with barium titanate nanostructures to induce a piezoelectric effect via external US stimulation. The authors further aim to create a hybrid platform that integrates electrical stimulation with the controlled release of NGF. This is accomplished by incorporating NGF within a thermoresponsive poly (N-isopropylacrylamide) (pNIPAM) hydrogel, which is applied to the exterior of the piezoelectric nanofibers. The *in vitro* characterization employed a PC12 cell model to evaluate the scaffold’s capacity to induce neuronal differentiation and promote axonal extension. The findings demonstrate that the combined effect of released NGF and piezoelectricity significantly enhances the differentiation of PC12 cells into neurons. Nevertheless, no statistically significant differences were noted between the group receiving only NGF release and the group subjected to both drug release and piezoelectricity. A similar trend was observed in axonal elongation assays: the combined treatment exhibited significant differences compared to the control, but not in comparison to the group with only NGF release. These results indicate a predominant role of NGF in modulating specific cellular functions of PC12 cells, compared to the influence of piezoelectricity. The *in vivo* validation studies conducted on the rat sciatic nerve corroborate the trends observed *in vitro*, indicating that NGF predominantly facilitates nerve regeneration compared to piezoelectric stimulation. Despite this, the motor recovery results are comparable to those achieved with autografts, underscoring the significant potential of this strategy for enhancing nerve regeneration. However, it is noteworthy that for all the cited studies the reinnervation experiments were performed using a non-critical gap (10 mm), which limits the generalizability of the findings. To fully assess the efficacy of this approach, it should be tested on larger gap sizes beyond the LGL, as such conditions are more likely to benefit from this strategy. It is also pertinent to address the ongoing debate regarding the effectiveness of US as a standalone approach for supporting nerve regeneration. While certain studies have demonstrated the potential of low-intensity pulsed ultrasound (LIPUS) in animal models—showing benefits such as enhanced axonal myelination, reduced expression of inflammatory cytokines, and supportive effects on SCs activity (Ito et al., 2020; Acheta et al., 2022)—the overall utility of LIPUS alone in facilitating nerve repair remains contentious. Furthermore, questions concerning the efficacy and safety of LIPUS in reliably triggering action potentials are yet to be conclusively resolved (Rodríguez-Meana et al., 2024).

#### 2.3.2 Plasmonic nanoparticles

**FIGURE 6:**
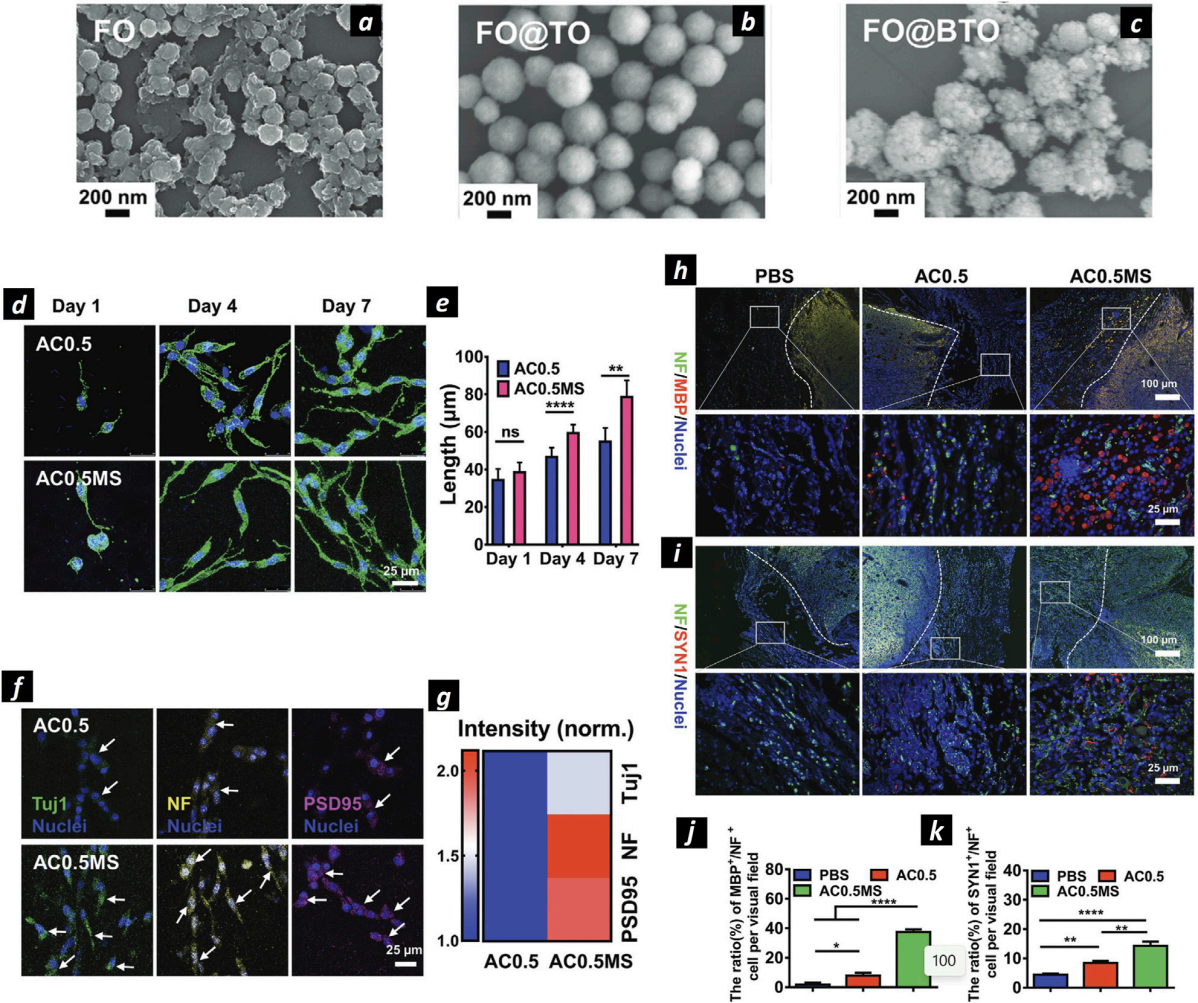
Magnetoelectric nanoparticles for neural regeneration. SEM images of SPIONs NPs **(A)**, SPIONs@TiO_2_ **(B)**, and SPIONs@BaTiO_3_ **(C)**. Confocal microscopies of PC12 cells stained by phalloidin (green) and DAPI (blue) **(D)**. The statistical cell length of PC12 cells cultured in AC0.5without (AC0.5) or with magnetic field stimulation (AC0.5MS) (n ≥ 60) **(E)**. Confocal microscopies of PC12 cells stained with Tuj1 (green), NF (yellow), PSD95 (magenta), and nuclei (blue), respectively **(F)**. Heating map of normalized fiuorescent intensity of three types of proteins **(G)**. Immunofiuorescence images of NF/MBP/Nuclei **(H)** and NF/SYN1/Nuclei **(I)** stained transected spinal cords. Quantification of the proportion of MBP+/NF+ **(J)** and SYN1+/NF+ **(K)** cells. Adapted and reproduced with permission of (Zhang et al., 2021). Copyright Wiley & Sons.

Heat conversion upon light shining to noble metal NPs (gold, silver, platinum) has grown interest in the scientific community to fabricate targeted nanotransducers for biomedical applications. Upon precise geometrical and dimensional tuning, gold nanostructures, such as nanospheres, nanoshells, nanorods, and nanostars can convert the absorbed light energy into heat via a process called localized surface plasmon resonance (LSPR) (Hutter and Fendler, 2004). This phenomenon has found interest in the Scientific Community, and successful applications in surface-enhanced Raman scattering (SERS) detection of biomolecules (Langer et al., 2020), and in the targeted treatment of cancerous lesion via photothermal effect were characterized in the recent years. In a pioneering work by the group of Jennifer West, 110 nm diameter gold nanoshells were used to destroy tumor mass *in vivo* with thermal ablation upon intravenous injection of NPs in mice and subsequent near-infrared (NIR) light excitation of LSPR (Hirsch et al., 2003). Nanoshells were synthesized to selectively adsorb NIR light (λ = 820 nm), due to scattering-limited attenuation and minimal heating of NIR light (650–950 nm) for biological tissue, which allows light penetration beyond 1 cm of depth. For this reason, this range of light is called a biological window (Sun et al., 2020). In recent years, multiple works have been published in this field, regarding materials characterization and advances toward the clinical application of such a promising anti-cancer technique (Redolfi Riva et al., 2014; 2017; Yang et al., 2019; Chen J. et al., 2020). Recently, localized gold NPs heating upon NIR photons absorption was also exploited in the field of neuroscience, mainly to induce modulation of neurons action potential (Figure 6_1).

In this framework, action potential stimulation was demonstrated upon visible light irradiation of gold nanorods incubated with rat’s dorsal root ganglion cells (Carvalho-de-Souza et al., 2015). Here, Ts1, a ligand that binds voltage-gated sodium channels and antibodies that target TRPV1 and P2X3 ion channels expressed by DRG neurons, were conjugated to AuNPs using biotin-streptavidin interaction, thus enhancing particles grafting to the cell membrane. Moreover, a pulsed green light laser (λ = 532 nm, power = 31 kW/cm^2^) was irradiated to the cells, and action potentials were recorded with the patch-clamp technique. Ligand grafting of AuNPs was crucial for stimulation, as it allows cell-particle proximity and guarantees tight contact between them, as was demonstrated by washing procedures that were unable to detach AuNPs from the DRG membrane. Interestingly, cell stimulation was dependent on the rate of temperature rise, not on the temperature value itself. This effect was also shown by IR irradiation of nerves that was able to both stimulate and inhibit action potential by changing the temperature rise: strong and rapid temperature gradients enabled neurons stimulation, whereas slow and controlled temperature rise triggered axon blockage (Wells et al., 2005; Lothet et al., 2017; Ganguly et al., 2019). Photoactivated AuNPs were also demonstrated to inhibit axon potential firing. Gold nanorods (AuNRs) and nanostars were incubated with mouse hippocampal neurons culture and irradiated with NIR light to induce NPs-mediated photothermal heating near the cell (Eom et al., 2014; Yoo et al., 2014) (Figure 5_1a). Cell membrane heating was possible thanks to the attachment of gold nanorods (GAuNRs) (Figure 5_1b) to the phospholipidic cell membrane (Figure 5_1c and 5_2d). The electrical activity from the cultured neuronal networks incubated with AuNRs was recorded with MEAs (Figure 5_1e), subsequently inhibited upon NIR irradiation and fully restored when NIR light was removed (Figure 5_1f and 5_1g). Moreover, the suppression of action potential was possible even when neurons were treated with a synaptic blocker, indicating that photothermal stimulation interfered with the generation of action potentials (Figure 5_1h and 5_1i). The authors reported that heat-mediated activation of the TREK-1 channel was responsible for action potential inhibition, as parallel investigation using fluoxetine (TREK-1 channel blocker) showed the absence of action potential inhibition during NIR light shining (Yoo et al., 2014). Laser intensity and timing could effectively modulate the degree of action potential inhibition, providing a promising nanotechnological platform to induce wireless neuronal modulation. However, the mechanism behind action potential suppression upon cell membrane heating has yet to be elucidated, as the same process could also be used to stimulate nerve action potential *in vivo* upon NIR laser heating with different laser parameters (Eom et al., 2014).

Localized temperature increase via photothermal effect has also been demonstrated to enhance neuronal cells viability, axons sprouting, and SCs proliferation as reported by evidence on rodent model. These studies suggested that laser phototherapy can induce wound healing, boost cell metabolism, enhance myelin production and attenuate pain (Bagis et al., 2002; Kate et al., 2018; Xu et al., 2022). The underlying mechanism behind enhanced nerve repair upon phototherapy has yet to be fully understood, but recent reports suggest selective temperature rise could induce SCs proliferation when coupled with NGC implant (Liu et al., 2014) and metabolism (Anders et al., 2004). As an example, SCs culture irradiation with He-Ne laser showed SCs proliferation enhancement in a dose-dependent manner (Van Breugel and Bär, 1993) and 810 nm diode laser (50 mW with two different energies: 1 J/cm^2^ and 4 J/cm^2^ for 32 s for 3 consecutive days) was able to increase cell proliferation after the seventh days of observation and to enhance neurotrophic factors production after 20 days from the treatment interruption. This direct *in vitro* evidence highlighted that phototherapy might have a role in inducing SCs proliferation and regulating mRNA expression associated with neuroprotein production (Yazdani et al., 2012) and represent a consistent indication in favor of its use for post-traumatic or post-surgical nerve repair. Not only SCs, but neuronal cells also have been proven to be sensitive to phototherapy, as previous reports evidenced increased axon sprouting and neurite outgrowth as a consequence of laser stimulation (780 nm for 1, 4, or 7 min) of neurons isolated by rats’ brain (Rochkind et al., 2009). The authors postulated that the possible mechanism of photo-induced axon sprouting is the upregulation of calcitonin gene-related peptide (CGRP) mRNA expression. Hence, by modulating the intensity or temporal pattern of injury-induced CGRP expression, phototherapy could increase the rate of regeneration and neuronal survival (Snyder et al., 2002).

Considering these encouraging results on cell experiments, *in vivo* studies on the feasibility of phototherapy to effectively enhance nerve regeneration have been conducted to validate the reported evidence. In this regard, low-level laser therapy (LLL) with 660-nm GaA-lAsP laser diodes (50 mW) was used to irradiate transcutaneously for 2 min daily for 10 consecutive days the injured site of rats’ sciatic nerve bridged with a synthetic NGC (Shen et al., 2013). Results confirmed that phototherapy was able to enhance motor function, and muscle reinnervation and reduce muscle atrophy compared to non-treated animals, demonstrating the feasibility of LLL to accelerate nerve repair of a 15-mm transected rats’ sciatic nerve. Numerous animal studies reported in a review by the group of Shimon Rochkind (Gigo-Benato et al., 2005) demonstrated the feasibility of phototherapy in enhancing nerve regeneration after axotomy thanks to various postulated mechanisms, such as increased cell metabolism, growth-associated protein-43 (GAP-43) expression and by reducing the degenerative state in spinal cord neurons, after laser shining (Rochkind et al., 1990). Hence, a dual positive effect of phototherapy for nerve regeneration could be evinced by shining light in correspondence of the spinal cord neurons and the site of the lesion, thus inducing neuron axon sprouting from the soma and consequently enhanced myelination and distal stump reinnervation due to the previously suggested effect of light to SCs.

In order to push forward all this encouraging evidence regarding the use of light to accelerate nerve regeneration, we envision the synergic use of a laser with nanostructured plasmonic nanoparticles that would allow us to precisely target the photothermal effect to the desired cells and finely modulate the generated heat. In this regard, the employment of NIR-adsorbent plasmonic nanostructures could be advantageous due to the high penetration of near-infrared photons and the feasibility of controlling the spatial orientation of NPs in order to target the generated heat selectively. Further efforts in material science will enable this possibility. Preliminary *in vitro* results demonstrated that laser activation of AuNRs can induce neurite outgrowth (Paviolo et al., 2013) (Figure 6_2). In particular, poly (styrene sulphonate) (PSS)-coated and silica-coated AuNRs (Figure 6_2a-c) were incubated with NG108-15 neuronal cells. Fluoresence images confirmed successful AuNRs cell encapsulation within neuronal cells that were homogeneously dispersed within cytoplasm without entering the nucleus (Figure 6_2d-f). Further cell irradiation with 1.2–7.5 W/cm^2^ laser at 780 nm wavelength, reported a 36% increase in neurite length for laser irradiated NPs respect to non-irradiated ones (Figure 6_2g). The higher irradiation dose (7.5 mW/cm^2^) showed no damage to the cellular structure, preserving its viability. In summary, the authors speculated that the observed behavior was not related to the AuNRs surface chemistry, indicating that increased neurite length resulted solely from photothermal conversion upon laser stimulation of the nanoparticles. A more recent study reported fabrication of NGF-functionalized core/shell iron oxide/Au nanoparticles (NGF-IrOx@Au NPs). PC-12 neuron-like cells were subjected to irradiation with LEDs (525 nm) at intensities of 1.09, 1.44, and 1.90 mW/cm^2^. A pronounced Ca^2+^ influx was detected in differentiated PC-12 cells treated NPs and irradiated at 1.44 and 1.90 mW/cm^2^, with cell viability exceeding 84% and notable proliferation observed. Cells exposed to both NGF-IrOx@Au NPs and 1.90 mW/cm^2^ LED exhibited a substantial increase in neuronal differentiation (83%) and neurite outgrowth (51%). Furthermore, upregulation of the neural differentiation marker β3-tubulin and the cell adhesion molecule integrin β1 was confirmed via RT-PCR and Western blot analysis (Yuan et al., 2019).

Although the encouraging evidence, the mechanism behind increased neurite outgrowth upon laser irradiated AuNRs is not yet understood, and more studies are needed to acquire evidence of the beneficial effect of the combination of AuNPs and NIR laser for neural regeneration and differentiation. As an example, other wavelength of the NIR physiological window should be tested, as it is already reported in literature that the 780 nm wavelength possess a stimulatory effect *per se*, as this wavelength is absorbed by cell mitochondria and can activate the NF-kB pathway, which is involved in various cell processes, as synaptic plasticity (Albensi and Mattson, 2000).

#### 2.3.3 Magnetic nanoparticles

Superparamagnetic iron oxide nanoparticles (SPIONs) have attracted strong attention in the last decades due to their well know properties of high magnetic susceptibility like ferromagnetic structures, almost absent magnetization residual once the external magnetic source is removed, very small size (d ≈ 10–20 nm) and safety (FDA-approved for medical use) (Xu and Sun, 2013). This class of NPs has been widely used in the biomedical field as drug delivery systems (Grillone et al., 2015; Redolfi Riva et al., 2020), MRI contrast agents (Avasthi et al., 2020), and to destroy cancerous mass by inducing thermal-mediated apoptosis (magnetic fluid hyperthermia) (Wu et al., 2019).

Recently, some works identified these NPs as candidates to enhance nerve regeneration, as demonstrated by the group of Tai Hyun Park, showing that SPIONs internalized by PC12 cells and exposed to NGF could enhance axon sprouting with a synergic effect, via activation of the mitogen-activated protein kinase (MAPK) signaling pathway (Kim et al., 2011). The authors showed a good yield of synthesized PEG-coated MNPs, with safe internalized concentration up to 20 μg/mL as evidenced by MTT assay and postulated that Fe^3+^ ions released by the particles in the cytoplasm might be responsible for neurite elongation. Moreover, exploiting the possibility of magnetically tracking SPIONs under the effect of an external magnetic field, some recent studies showed the possibility of directing PC-12 neurites’ extension and orientation upon internalization via the TrkA receptor and subsequent magnetic manipulation. This process could induce micromechanical stresses of the order of pN that were responsible for axons growth (Riggio et al., 2014). For instance, PC12 cells labeled with 25 nm PEI-coated SPIONs showed increased neurite and axon length under a magnetic field, which also enhanced nerve cell differentiation. The synergy between PEI-coated SPIONs and the magnetic field raised the orientation index from 0.006 to 0.57. A relevant study that explored the possibility to orientate axonal growth with the applied magnetic field exploited SPONs internalized within PC12 and spiral ganglion neurons (SGNs) (Hu et al., 2021). Under the effect of 80–90 mT external magnetic field the authors proved enhanced neurite outgrowth of PC12 cells. Interestingly, when SPIONs were internalized within SGNs under the same experimental conditions promoted the development of growth cones in the same direction of the applied magnetic field, as shown by anti β-tubulin and phalloidin staining of actin-supported growth cone. This effect is particularly interesting for treating neurotmesis, as it helps prevent misdirected axonal growth and the resulting reinnervation to incorrect targets. Marcus et al. further demonstrated that SPIONs under a magnetic field have a higher interaction with the PC12 cell membrane, affecting the migration pattern and allowing control over cell distribution. Additionally, the magnetic field influenced neurite growth direction, predominantly toward the field source, with slight growth detected in the opposite direction, possibly aiding in cellular mechanical support. SPIONs were also used to target magnetically doped adipose-derived mesenchymal stem cells to regenerate rat’s sciatic nerve and to dope electrospun multilayers NGCs to allow mechanical reinforcement and sustainable delivery of melatonin, that has been proven to enhance nerve regeneration *in vivo* (Chen X. et al., 2020). Immunohistochemical analysis revealed a higher quantity of SCs and neurofilaments for both melatonin-loaded NGCs (ML-NGCs) and SPIONs-loaded NGCs with respect to bare PCL NGCs, displaying that both the loaded components were able to enhance nerve regeneration. SPIONs properties were also recently combined with piezoelectric materials in a core@shell structure to exploit magnetically triggered ES (magnetoelectric effect) (Figure 6). Core@shell SPIONs@BaTiO3 NPs (Figures 6A–C) were synthesized and used for this study. The wireless transmission of electrical signals was achieved by coupling the magnetic effects of SPIONs in a magnetic field (13 mT, 60 Hz) with the piezoelectric properties of BaTiO_3_. It was found that the combination of SPIONs@BaTiO_3_ nanoparticles with magnetic field-electric stimulation significantly enhanced PC12 cell proliferation and neurite length compared to magnetic field stimulation alone (Figures 6D, E). Immunohistochemistry further confirmed the differentiation of PC12 cells, as evidenced by the increased expression of neuron-specific proteins, such as Tuj1, NF (which regulates axon diameter), and PSD95 (indicative of synaptic structure) (Figures 6F, G). The study also reported that the higher expression of L-VGCC ion channels and increased Ca^2+^ release induced by the magnetoelectric stimulation in SPIONs@BaTiO_3_ NPs was the primary mechanism driving PC12 cell differentiation (Zhang et al., 2021). The study further revealed that magnetoelectric grafts, responsive to external magnetic fields, can generate electrical signals via the magnetoelectric effect, thereby facilitating the regeneration of functional axons capable of forming synapses and myelin sheaths. This is evidenced by the immunofluorescence images and corresponding quantitative analysis. The ratio of MBP+/NF + cells per visual field was lowest in the PBS group (1.8% ± 1.3%), increased to 7.87% ± 1.9% in the AC0.5 group with composite hydrogel implantation, and significantly elevated to 37.5% ± 1.8% in the AC0.5MS group. Similarly, immunofluorescence images (Figures 6H, I) and quantitative data (Figures 6J, K) indicated a markedly higher expression of SYN1 in the AC0.5MS group compared to the other groups. Interestingly, SPIONs were also used in another recent study in combination with antegrade neural tracing imaging for the peripheral nervous system. The authors used Fe_3_O_4_@COOH nanoparticles conjugated with biotinylated dextran amine (BDA, a neural tracer) to generate antegrade nano-neural tracers (NNTs). These tracers are encapsulated in microfluidic droplets of GelMA to mitigate leakage and ensure a controlled, sustained release. NNTs were able to successfully monitor neural regeneration in real time using MR/PAI dual real time imaging modality (Ren et al., 2022).

All this evidence clarified the beneficial role of nanostructured materials to induce a stimuli-responsive effect to enhance nerve regeneration via the application of an exogenous stimulus converted into mechanical, thermal, or electrical energy. Such a scenario allows the envisioning of a new therapeutic strategy based on nanotransducers NGC functionalization and subsequent wireless external stimulation to accelerate nerve regeneration and to improve functional recovery over the critical gap length and for those lesions characterized by huge distances between the damaged site and the target muscles to reinnervate, such as brachial plexus lesions (Bianchini et al., 2023b). Further studies will be needed to understand better the biochemical pathways triggered by such stimuli involved in enhanced reinnervation and to clarify the safety of inorganic NPs implantation.

## 3 Outlook and future perspectives

**FIGURE 7:**
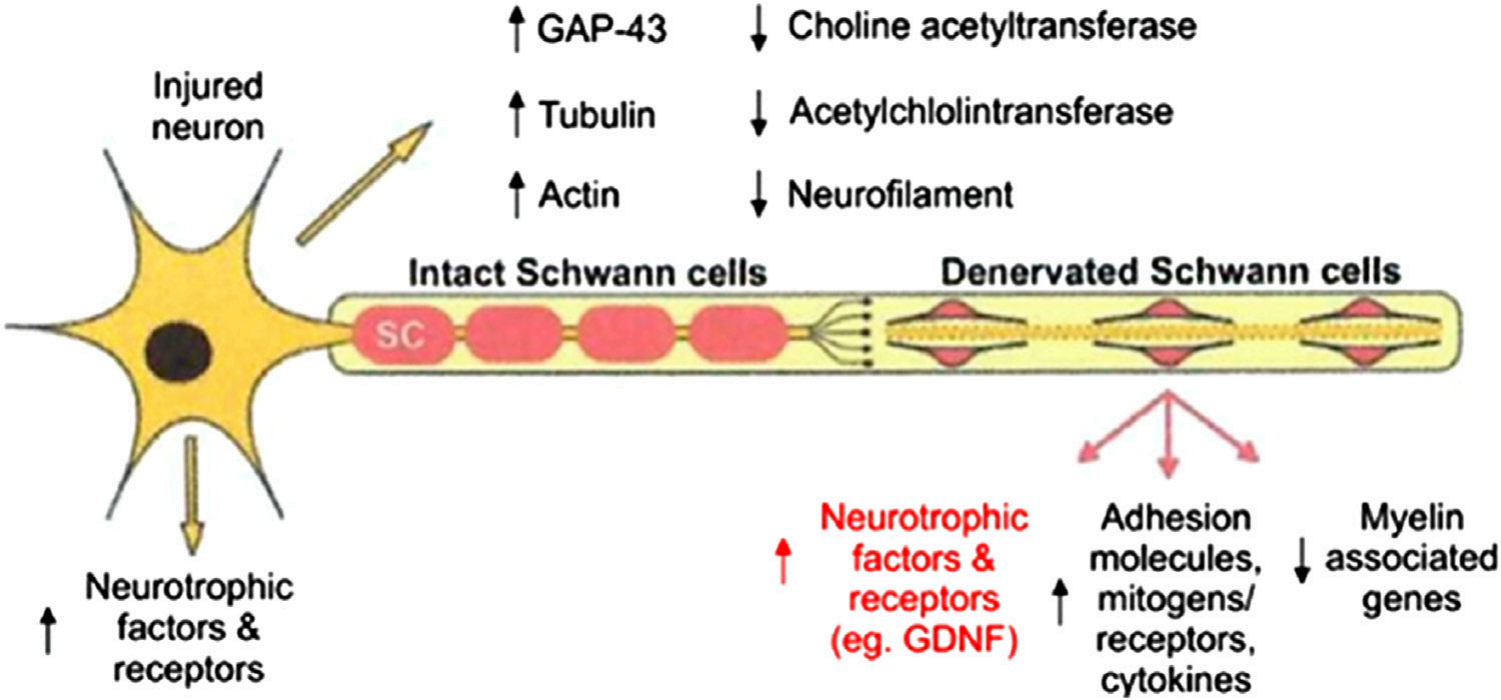
Schematization of growth-associated genes expressed in axotomized motoneurons after target loss and in chronically denervated SCs in the distal nerve. Reproduced with permission from (Gordon, 2020). Copyright MDPI.

The repair of damages beyond the LGL represents the most significant challenge in the field of peripheral nerve regeneration. It involves a complex network of pathways activated across different cell types, whose synergistic roles ultimately determine the success or failure of nerve regeneration (Deumens et al., 2010; Riva et al., 2024). After neurotmesis, calcium signaling, mediated by the phosphorylation of protein kinase (MAPK) and Ca^2+^/calmodulin-dependent protein kinase (CaMK), initiates retrograde signaling pathways that upregulate injury-responsive gene expression in the neuronal soma, thereby facilitating axonal outgrowth. Meanwhile, in the distal stump denervated SCs undergo differentiation, proliferation, and elongation while downregulating the expression of myelin-associated genes and upregulate growth-associated genes (Figure 7) (Gordon, 2020).

Facilitating and accelerating these cellular processes is crucial for repairing LGLs, where partial sensory recovery may be achieved, but motor recovery is often limited due to the substantial distance between the injury site and the target muscle. This gap can result in the distal nerve stump’s inability to sustain regenerating axons, leading to fibrotic changes in the endoneurial tubes and a loss of SC pro-regenerative properties, including neurotrophin production and growth-supportive phenotypes (Fu and Gordon, 1995). This regression is linked to a progressive decline in regenerative efficacy following delayed nerve repairs or proximal nerve injuries, which require extensive nerve regeneration over long distances to reinnervate distant denervated target organs, such as brachial plexus lesions. This leads to only partial recovery of motor function in the denervated limb, resulting in permanent loss of mobility and significant impact on the patient’s quality of life. Over the past 3 decades, various therapeutic strategies have been explored to address this challenge (Table 1).

**TABLE 1:** Summary of strategies to enhance peripheral nerve regeneration.

Strategy	Typology	Advantages	Limitations	References
Surgery	End-to-end neurorrhaphy	• High degree of functional recovery • Low risk of fascicles misalignment	Only for short nerve damages	Kline (1990), Pfister et al. (2011)
Nerve graft	Autograft	• Good degree of functional recovery in limiting gap lesions	• Dimensional mismatch • Low availability • Donor morbidity	Ray and Mackinnon (2010)
	Decellularized nerve graft	• Good availability • Good degree of functional recovery in limiting gap lesions	• Decellularization methods impair physiochemical properties of the graft	
Engineered NGCs		• High availability • Highly customizable to target nerve features • Possibility to integrate several functions (controlled drug release, stimuli-responsive nanomaterials	• Poor functional recovery in limiting gap lesions • Need to find strategies to support regeneration over the LGL	Riva et al. (2024)
Drug delivery	Neurotrophins (NGF, BDNF, GDNF, NT-3, NT-4)	• Promote survival and growth of both sensory and motoneurons • Support neurite regrowth • Induce neuronal differentiation of progenitor cells • Stimulate neurogenesis	• Poor stability (especially neurotrophins) • Need to find strategies to reduce burst release • Difficulties to sterilize the conduit prior implant	Manoukian et al. (2020), Bianchini et al. (2023a)
	4-aminopyridine (4-AP)	• K+ channels inhibitor • Support neurite regrowth		
	Erythropoietin (EPO)	• Neuroprotective action • Anti-inflammatory		
	Tacrolimus (FK506)	• Immunosuppressant • Neuroprotective action • Support neurite regrowth		
Cell therapies	Mesenchymal stem cells (MSCs)	• Can be differentiated into SCs • Release neurotrophins • Good proliferation capacity, easy to handle	• Safety issues due to their proliferation capacity (for MSCs and iPSCs) • If seeded within a device, difficulties in sterilization procedures • Need for a structural support to avoid apoptosis and support their growth • Lack of regulatory approval for clinical translation	Lackington et al. (2017), Kubiak et al. (2020)
	Induced pluripotent stem cells (iPSCs)	• Can differentiate into any cell type		
	Autologous SCs	• Non immunogenic • Enhance nerve regeneration		
Stimuli-responsive nanostructured materials	Conductive polymers	• Biocompatible • Possibility to tune geometrical and physiochemical properties	• Difficult to localize ES • Need for cables and stimulators to deliver ES	This work
	Graphene family materials	• Selectivity to localize ES • Good electrical properties	• Safety concerns for long term toxicity (mainly CNTs)	
	Piezoelectric nanoceramics	• Selectivity to localize ES • Possibility to activate ES in wireless modality • High cytocompatibility *in vitro*	• Concerns on long-term cytotoxicity. Although studies demonstrated high cytocompatibility *in vitro* and the absence of tissue toxicity *in vivo* for few weeks, studies with follow-up at least up to 1 year are needed to ensure clinical translation of these approaches	
	Plasmonic nanoparticles	• High tunability of photothermal properties • Possibility to exploit tissue penetrable NIR light		
	Magnetic nanoparticles	• Received FDA approval for clinical use • Possibility to exploit magnetoelectrical conversion for wireless ES • Can guide direction of axonal regrowth		

Epineural suturing remains the preferred technique in clinical practice due to its superior functional recovery rates; however, it is often unsuitable for large lesions that prevent direct suturing of nerve stumps. Engineered NGCs were mentioned in the Introduction as a promising strategy for nerve damage repair and the restoration of lost sensorimotor functions. However, their limited success in repairing limiting gap lesions has driven the scientific community to explore solutions aimed at enhancing this technology and making it a reliable clinical alternative to autografts. The employment of drug delivery systems for the controlled administration of nerve growth factors and the functionalization of NGCs with proteins, peptides, and stem cells are the most extensively discussed strategies. For a comprehensive discussion of these aspects, reference is made to the works by (Bianchini et al., 2023a; Wieringa et al., 2018). However, although some of these strategies have been validated *in vivo* in rodent models, none have yet received regulatory approval for clinical use. Specifically, nerve growth factors are susceptible to degradation and burst release, thus failing to adequately supply the NGC luminal environment with a sustained molecule concentration necessary to facilitate the reinnervation process of the gap (at least 1 month for injuries beyond the LGL). Similarly, regulatory approval is lacking for stem cell therapies, despite the promising results observed in small animal models from studies conducted on NGCs loaded with autologous SCs, neural stem cells, and bone marrow-derived stem cells (Lackington et al., 2017).

As a result, research in this field is shifting towards investigating the use of external physical stimuli—such as electrical, thermal, mechanical, and magnetic fields—to enhance nerve regeneration. When precisely controlled by their respective delivery devices, these stimuli provide reliable means to address the limitations of growth factor treatments and cell-based therapies. To date, the acute application of low-frequency ES (20 Hz, 20–60 min) during neurorrhaphy in rodent model showed promise by enhancing nerve regeneration, accelerating axonal regrowth, and reducing the time required for reinnervation of the target muscle (Willand et al., 2016). Despite its promising results, this technique is constrained by the use of intraoperative electrodes, which can extend surgical duration and potentially lead to long-term issues such as infections and inflammation. Additionally, the technique has proven effective only for medium-sized lesions (2–4 cm). Many peripheral nerve injuries, unfortunately, involve much greater distances between the lesion site and the target muscles (approximately 8–10 cm on average for brachial plexus injuries). ES is currently not employed in clinical practice to improve peripheral nerve regeneration. Instead, its applications are limited to pain management and the induction of muscle contractions (functional ES). Consequently, research has focused on developing more advanced and precise solutions eventually delivered in wireless modality, thereby avoiding the long-term inflammatory complications associated with traditional methods. The purpose of this Review aimed to gather the most relevant studies concerning the combined use of external physical stimuli and nanostructured biomaterials for to trigger specific cellular functions that can support and accelerate nerve regeneration process in limiting gap lesions. This combination is emphasized due to its potential to markedly enhance the selectivity and precision of the physical stimulus, confining its effects to the targeted cells (Micera and Redolfi Riva, 2022; Ciofani et al., 2023). This approach ensures the modulation of cellular functions exclusively within the target area, minimizing the impact on adjacent tissues. The application of physical stimuli without the integration of nanomaterials is discussed in (Wei et al., 2023). Among the studies reviewed, those utilizing ceramic nanostructures that convert mechanical stimuli, transmitted via ultrasound, into localized electrical stimulation through the piezoelectric effect appear particularly promising. This approach offers several advantages: it operates wirelessly, thus avoiding the need for cables and implantable electronics, and enables precise localization of the electrical stimulus. The small size of the employed piezoelectric materials allows direct targeting of cellular structures, ensuring the precision needed to trigger the biochemical functions associated with regenerative processes. Moreover, the advanced manufacturing techniques enable the customization of shape and geometry of carrier materials. This flexibility allows these biomaterials to move beyond the traditional tubular design of NGC, taking on alternative forms such as injectable hydrogels, as recently proposed by (Li et al., 2024) in the framework of spinal cord injury repair. Although laser therapy showed promise to support nerve regeneration, photothermal conversion of near-infrared (NIR) light by plasmonic nanostructures to localized temperature increases still seems to be far from providing reliable solutions in regenerative medicine. However, recent advancements in photothermal neuromodulation are noteworthy. The promising results reported by Yoo and colleagues (Yoo et al., 2014) are particularly significant, as, if validated in animal models, they could lead to an innovative and selective technique for modulating action potentials in peripheral nerves, potentially eliminating the need for implantable stimulators and electrical cables. research involving superparamagnetic iron oxide nanoparticles (SPIONs) shows promise too, as these materials have been shown to promote axonal elongation in the direction of an externally applied magnetic field *in vitro*. Their potential is enhanced by their FDA approval, which is due to their low toxicity and their ability to be excreted via renal clearance, owing to their small size (d < 20 nm) (Bobo et al., 2016).

All these solutions are of significant interest both for preclinical research and for a deeper understanding of the cellular biology involved in nerve regeneration.

However, to facilitate the clinical translation of stimuli-responsive nanostructures, it is crucial to address two key aspects:

1. The molecular mechanisms by which these nanomaterials support nerve regeneration.
2. The potential long-term toxicity of implanted nanomaterials.

**FIGURE 8:**
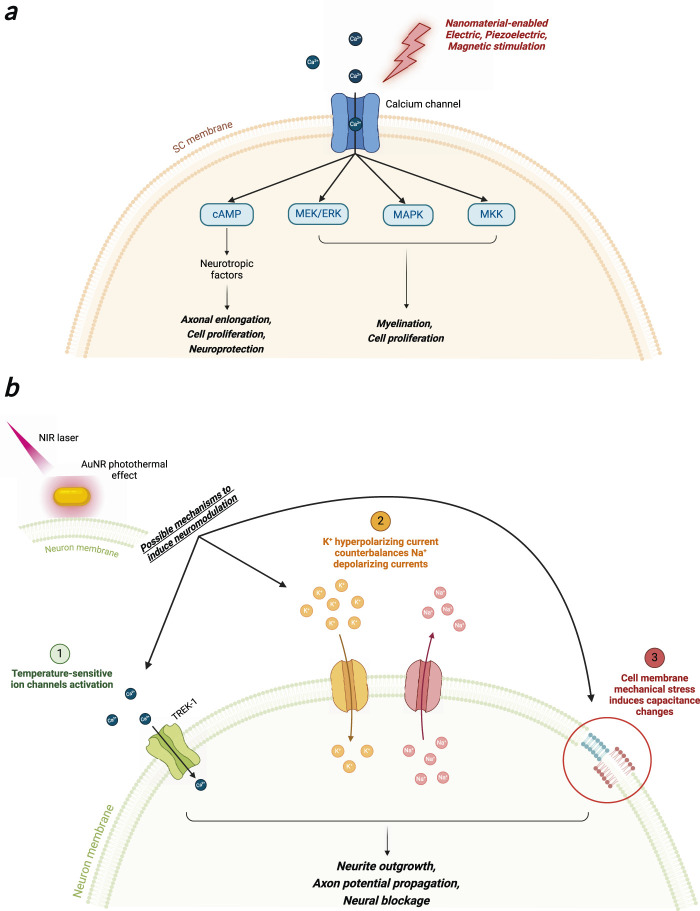
Possible cellular mechanisms that regulate neural function in regeneration and neuromodulation upon nanomaterial-enabled electric, piezoelectric, magnetic **(A)** and photothermal **(B)** stimulation. Created with Biorender.com.

This Review discussed some potential cellular pathways triggered by nanoatructured materials to induce nerve regeneration (Figure 8).

Specifically, external electrical, mechanical and magnetic stimuli induced a Ca^2+^ intracellular flux that triggered the activation of MAPK, MEK/ERK, and MKK pathways, that allow myelination, cell proliferation and axonal enlongation (Figure 8A). Additionally, activation of the cAMP pathway using piezoelectric stimulation promotes the expression of genes related to the production of neurotrophic factors, which facilitate axonal elongation. However, the complexity of these pathways poses several challenges. An emerging area of interest in peripheral nerve regeneration is the cross-talk between molecular pathways. Signaling pathways like PI3K/Akt, MAPK/ERK, and JAK/STAT do not operate in isolation; instead, they interact in a coordinated manner to ensure efficient repair. For instance, the PI3K/Akt and MAPK/ERK pathways are often co-activated by the same neurotrophic factors and share common downstream effectors, such as CREB (cAMP response element-binding protein), which influences gene transcription related to cell survival and growth. This review identified the MAPK/ERK and cAMP signaling pathways as key candidates that are upregulated by the combined influence of external physical stimuli and nanostructured materials in peripheral nerve regeneration, as demonstrated by several referenced studies. However, due to the intricate nature of cellular mechanisms, further investigation is required to elucidate whether there is potential crosstalk between distinct pathways or concurrent activation of multiple mechanisms, such as Trk receptors and neurotrophic factors. This would provide a clearer understanding of the complexity and interplay of the regenerative processes involved.

Action potentials modulation through the activation of temperature-sensitive channels such as TREK-1 was also reported (Yoo et al., 2014). Many studies suggest that more plausible mechanisms involve physical phenomena such as alterations in membrane permeability or the induction of hyperpolarizing K^+^ currents that counteract depolarizing Na^+^ currents, as well as membrane capacitance fluctuation due mechanical stress to the cell membrane in response to localized heating (Lothet et al., 2017; Ganguly et al., 2019) (Figure 8B). However, the mechanisms underlying the activation of cellular functions remain highly debated, and thus, further fundamental research is required to elucidate these phenomena.

Another crucial aspect to consider for the clinical translation of this strategy is the long-term safety of implanted nanostructured materials, mainly nanoparticles. An advantage of using nanoparticles is their small size, which allows them to deliver therapeutic effects near target cells or even within the cells themselves, through internalization into the cytoplasm. However, this capability has sparked considerable debate in the scientific community about the potential long-term cytotoxic effects of internalized nanoparticles (Soenen et al., 2015; Mohammadpour et al., 2019). Even though all the cited studies in this Review investigated the optimal nanomaterial concentration to exploit the desired effect of avoiding cytotoxicity to the cells, the possible long-term toxic effects of nanoparticles is still debated (Kunrath and Campos, 2021). A significant concern regarding internalized nanomaterials is their intracellular fate, as they can penetrate the nucleus and disrupt chromosomal integrity or interfere with cellular damage repair mechanisms, potentially leading to mutagenic effects (Sengul and Asmatulu, 2020). Moreover, interference with the protein synthesis chain could also induce mutagenesis. Due to their high surface area-to-volume ratio, nanoparticles exhibit increased reactivity and thermodynamic instability, which markedly increase the probability of interactions with cellular organelles, thereby altering essential cellular pathways. Over time, cell internalization of metallic nanoparticles may lead to chronic inflammation, oxidative stress with reactive oxygen species (ROS) production, and disruption of cellular functions, potentially increasing the risk of developing various diseases, including cancer, immune system and neurodegenerative disorders (Tee et al., 2016; Shukla et al., 2021). Various factors can influence or modify the toxicity of nanomaterials, including their physicochemical characteristics, such as size, shape, and material composition. Additional considerations include the presence of coatings to enhance colloidal stability, ligands for extracellular receptor targeting, their distribution method (either freely within the body or embedded in a biomaterial), and the route of administration (oral, intravenous, respiratory, or subcutaneous). A detailed discussion of these aspects can be found in (Mohammadpour et al., 2019; Huang et al., 2020; Sengul and Asmatulu, 2020). Several studies reviewed in (Sani et al., 2021) have investigated the toxicity of gold nanoparticles in murine models following a single intravenous injection. These particles exhibit size-dependent distribution across various body tissues: larger particles, over hundreds of nm, tend to remain in the bloodstream, while smaller particles, in the range of tens of nanometers, are cleared by the liver, spleen, and lungs. This accumulation has been associated with inflammatory processes within a few weeks of observation. Studies on magnetic nanoparticles (hydrodynamic diameter approximately 150 nm) have reported good biocompatibility, with these nanomaterials accumulating in the liver and spleen after 3 months of observation without evidence of toxic effects (Mejías et al., 2013). Smaller particles (50 nm) are capable of penetrating deeper into the body, including crossing the blood-brain barrier; however, the duration of these studies (4 weeks) is insufficient to assess potential long-term effects associated with this phenomenon (Kim et al., 2006). Ceramic nanoparticles are generally considered safe for biological systems due to their lower reactivity and chemical inertness, which prevents the release of toxic ions through oxidative processes—a phenomenon observed with metallic nanoparticles. Barium titanate particles, reviewed in this study, have been shown to be safe in cellular models, as also documented in (Genchi et al., 2016). However, long-term toxicity studies in animal models are still lacking to confirm the absence of potential toxic effects on organs (Sood et al., 2023).

Despite the complexity of the issue, current research on the long-term toxicity of nanoparticles remains limited, with few studies extending beyond 6–12 months of animal study. Most investigations focus on *in vitro* assessments or short-term studies in murine models, spanning only a few weeks to months. These time durations are inadequate for identifying potential long-term effects, especially for inorganic metallic or ceramic nanoparticles that, due to their small size (<20 nm), might remain in the body for years without degradation. Mohammadpour and colleagues recently investigated 1-year chronic toxic evaluation of a single intravenous injection of 100 mg kg^-1^ silica nanoparticles in female BALB/c mice (Mohammadpour et al., 2020). Although the study did not observe significant effects on the animals’ body weight or blood parameters, such as erythrocyte count and plasma biomarker levels, the rapid accumulation of nanoparticles in the liver triggered an inflammatory response, leading to microlesions detected in the liver, spleen, and lungs. The lack of long-term toxicity studies on nanoparticles is a significant concern, heightening apprehensions about their clinical translation. Only with further research and robust long-term safety data can we consider the clinical use of nanoparticles, thereby unlocking their remarkable therapeutic potential for peripheral nerve regeneration, as highlighted in various studies reviewed in this work. Conversely, non-internalized NPs that remain in contact with the implanted scaffold, or properly functionalized to avoid cell entrance, could be considered safer, as their cellular uptake could be drastically reduced. However, more efforts should be dedicated to clarifying this issue, as it represents one of the major bottlenecks of nanostructured materials for tissue engineering purposes.

Despite these considerations, this work has highlighted and discussed several promising strategies that utilize nanostructured materials activated by external stimuli to control cellular functions supporting nerve regeneration. Continued efforts by the Scientific Community to address the two key points discussed in this paragraph will provide critical data, potentially paving the way for the clinical application of these nanomaterials in peripheral nerve regeneration therapies.
